# The effect of caponization on tibia bone histomorphometric properties of crossbred roosters

**DOI:** 10.1038/s41598-024-54791-6

**Published:** 2024-02-19

**Authors:** J. Wojciechowska-Puchałka, J. Calik, J. Krawczyk, J. Obrzut, E. Tomaszewska, S. Muszyński, D. Wojtysiak

**Affiliations:** 1 Department of Animal Reproduction, Anatomy and Genomics, University of Agriculture in Kraków, 24/28 Mickiewicza Ave., 30-059 Cracow, Poland; 2 Department of Poultry Breeding, National Research Institute of Animal Production, 32-083 Balice, Poland; 3 Department of Animal Physiology, Faculty of Veterinary Medicine, University of Life Sciences in Lublin, 12 Akademicka St., 20-950 Lublin, Poland; 4 Department of Biophysics, Faculty of Environmental Biology, University of Life Sciences in Lublin, 13 Akademicka St, 20-950 Lublin, Poland; 5 Department of Animal Genetics, Breeding and Ethology, Faculty of Animal Sciences, University of Agriculture in Kraków, 24/28 Mickiewicza Ave., 30-059 Cracow, Poland

**Keywords:** Bones, Caponization, Collagen, Microstructure, Roosters, Tibia, Biological techniques, Cell biology, Zoology

## Abstract

The negative effect of caponization on the structural, geometric and mechanical parameters of femur and tibia has been shown in a few studies. Nevertheless, its influence on tibia bone microarchitecture is still largely unknown. Therefore, this study aimed to assess the effect of castration on the microstructural parameters of the trabecular and compact bone of tibia bone in crossbred chickens. The experiment involved 96 roosters derived from crossing Yellowleg Partridge hens (${\dot{\text{Z}}}$-33) and Rhode Island Red cockerels (R-11) fattened until the 16th, 20th and 24th week of life. Animals were randomly divided into 2 groups of 48 each. Group I (control) consisted of intact roosters and group II (experimental) consisted of birds subjected to caponization at the 8th week of age. The castration surgery had no influence on some properties within compact bone such as osteon diameter On.Dm, osteon perimeter On.Pm, osteon area On.Ar, osteocyte lacunar number Ot.Lc.N, osteon bone area On.B.Ar, osteon wall thickness On.W.Th as well as thick-mature collagen content in all analyzed age groups of animals. Nevertheless, our results demonstrate that castration caused a decrease of Haversian canal area Hc.Ar, osteocyte lacunar area Ot.Lc.Ar and osteocyte lacunar porosity Ot.Lc.Po among the 16-week-old birds, decrease of Haversian canal perimeter Hc.Pm and increase of fraction of bone area On.B.Ar/On.Ar among 16- and 24-week-old individuals and also an increase of osteocyte lacunar density Ot.Lc.Dn in the osteons of the oldest roosters. Additionally, some microstructural parameters of trabecular bone show the negative effect of caponization. The youngest 16-week-old capons were characterized by thinnin the trabecular in the epiphysis part of tibia. Moreover, in the case of 24-week-old, there is an increase in the trabecular separation Tb.Sp with simultaneous decrease of trabecular number Tb.N compared to roosters, which may suggest the increase of the bone resorption among the oldest individuals. The increased bone turnover in the epiphysis part of the tibia bone also indicates changes in the collagen fibers distribution, where among 20-week-old animals there is a decrease in the content of immature thin collagen fibers with simultaneous increase in the content of mature thick collagen fibers. Furthermore, among the oldest 24-week-old individuals we can observe the increased thick-to-thin collagen ratio, which may be a sign of slowing down in bone formation.

## Introduction

Poultry meat is the important part of the human diet. Unfortunately, the current progressive intensification of poultry production towards the increased muscle mass has contributed to a deterioration in the taste and dietary qualities of the meat. Hence, consumers are increasingly looking for quality products. Such conditions are fulfilled by capon meat from birds of native breeds^1–3^. Capon production is popular in countries such as the USA, Italy, France, Spain, China and Taiwan^1,4–7^. In Poland, native breeds, such as Greenleg Partridge or Yellowleg Partridge, are used to capons production. However, these animals achieve a small body weight. Therefore, it is common to use crossbreeding in order to obtain a higher weight of birds, while maintaining unique taste qualities^8^. Furthermore, studies on the effect of caponization on selected slaughter and meat quality traits confirm that capons have a higher body weight, higher breast muscle and leg weight compared to uncastrated birds^9,10^. However, an important criterion for selecting animals for capon production is not only body weight, but also the ability of the animals to adapt to uncomfortable environmental conditions as well as good resistance to disease.

Moreover, caponization is an option for poultry meat producers, since around 50% of roosters are obtained from laying hens^11–13^. The solution to these problems could be the caponization. The most common method of castration in European and Asian countries is the surgical removal of the testicles and epididymis^14,15^. This procedure is carried out before reaching sexual maturity^13,16^.

The loss of testicles results in changes of metabolic processes among birds^17,18^. Lack of sex steroids contributes to changes in behavior (lack of desire to mating, reduced aggressiveness) and appearance of birds (comb regression)^10,19,20^. Testosterone exerts anabolic effects in a variety of tissues, increasing protein synthesis and stimulating muscle and bone growth. The rearing period of capons is considerably longer than chicken broilers, hence they must have strong bones to support the growing body mass. What’s important is that the available literature also reports that a high ratio of muscle mass to bone mass leads to growth disorders, bone strain or even bone fractures^16,21,22^. Furthermore, some reports suggest that androgen deficiency in humans caused by various factors (age or testectomy surgery) inhibits bone growth and development. Therefore, some authors indicate that the castration procedure of birds impairs the development of long bones—their geometry and strength^16,21^. Nevertheless, negative changes are most often observed in the tibia bone, that is why it is the model bone in studies of growth, mineralization and strength of skeleton in birds^23^. However, one should remember that bone microstructure could be an important indicator of bone mechanical strength, regardlessly of bone mineral density or bone geometry^24^.

To our knowledge, there are a lack of experiments that show the effect of caponization on the microarchitecture of long bones among birds, but such analyses have been the subject of numerous studies among mammals^25–28^. Hence, the objective of this study was to investigate the effect of caponization on tibia bone microstructure, where the analyzed traits included assessment of trabecular and compact bone microarchitecture of tibia bone of crossbred birds. Applied techniques used in our previous work^29^ as well as in the present study can provide detailed information about mechanisms of bone tissue homeostasis among castrated roosters.

## Results

### Trabecular bone histomorphometric analysis

**Figure 1:**
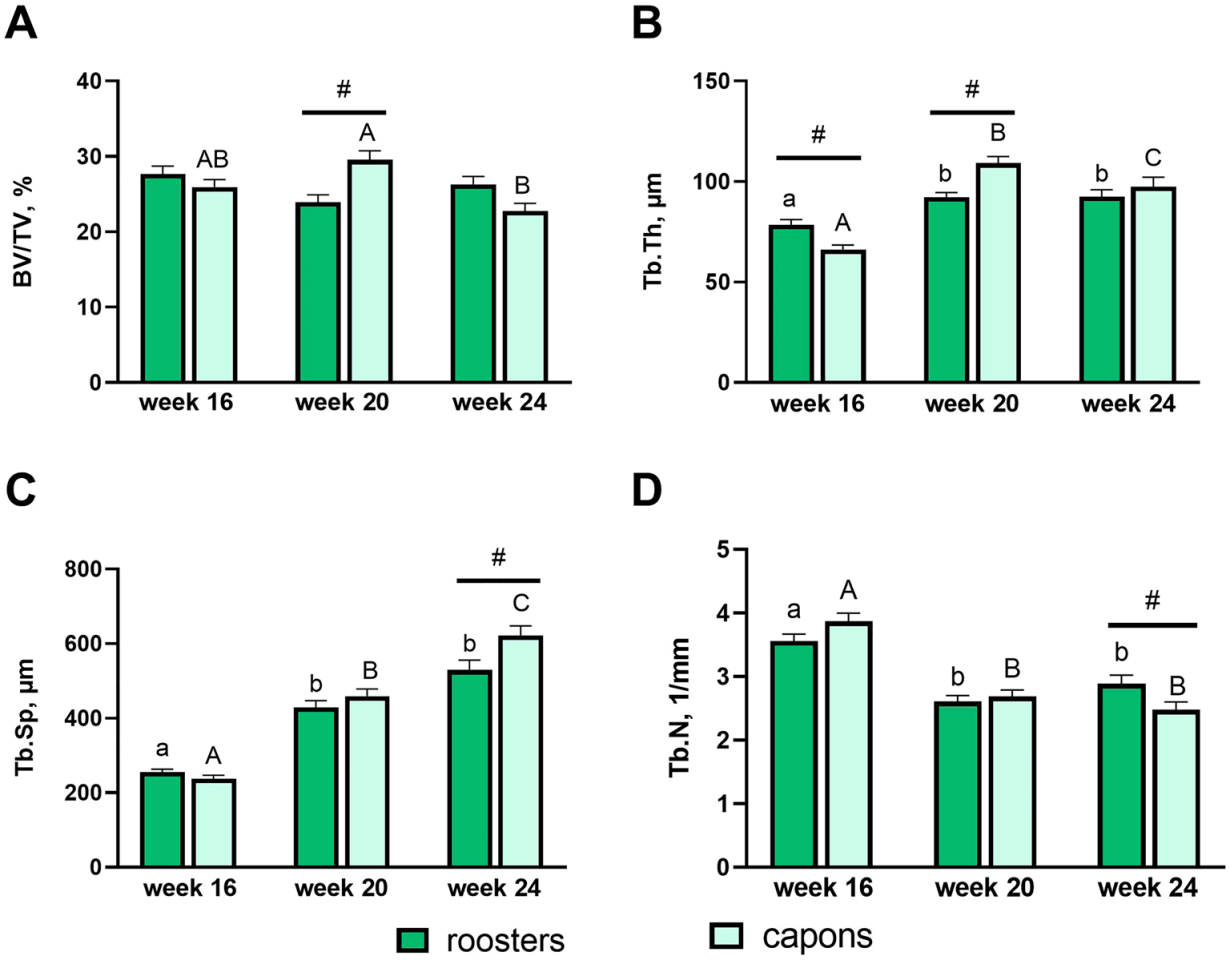
Histomorphometric parameters: bone volume/tissue volume (**A**), trabecular thickness (**B**), trabecular separation (**C**), trabecular number (**D**) of the trabecular bone in the epiphyses of tibia bone of 16-week-old, 20-week-old and 24-week-old roosters and capons. Data are presented as least squares means (LSM) and standard error of mean (SE), a, b, c—mean values between age groups within roosters with different letters differ significantly *p* < 0.05; (**A,B,C**) mean values between age groups within capons with different letters differ significantly *p* < 0.05; ^#^significant difference between capons and roosters within age (*p* < 0.05).

**Figure 2:**
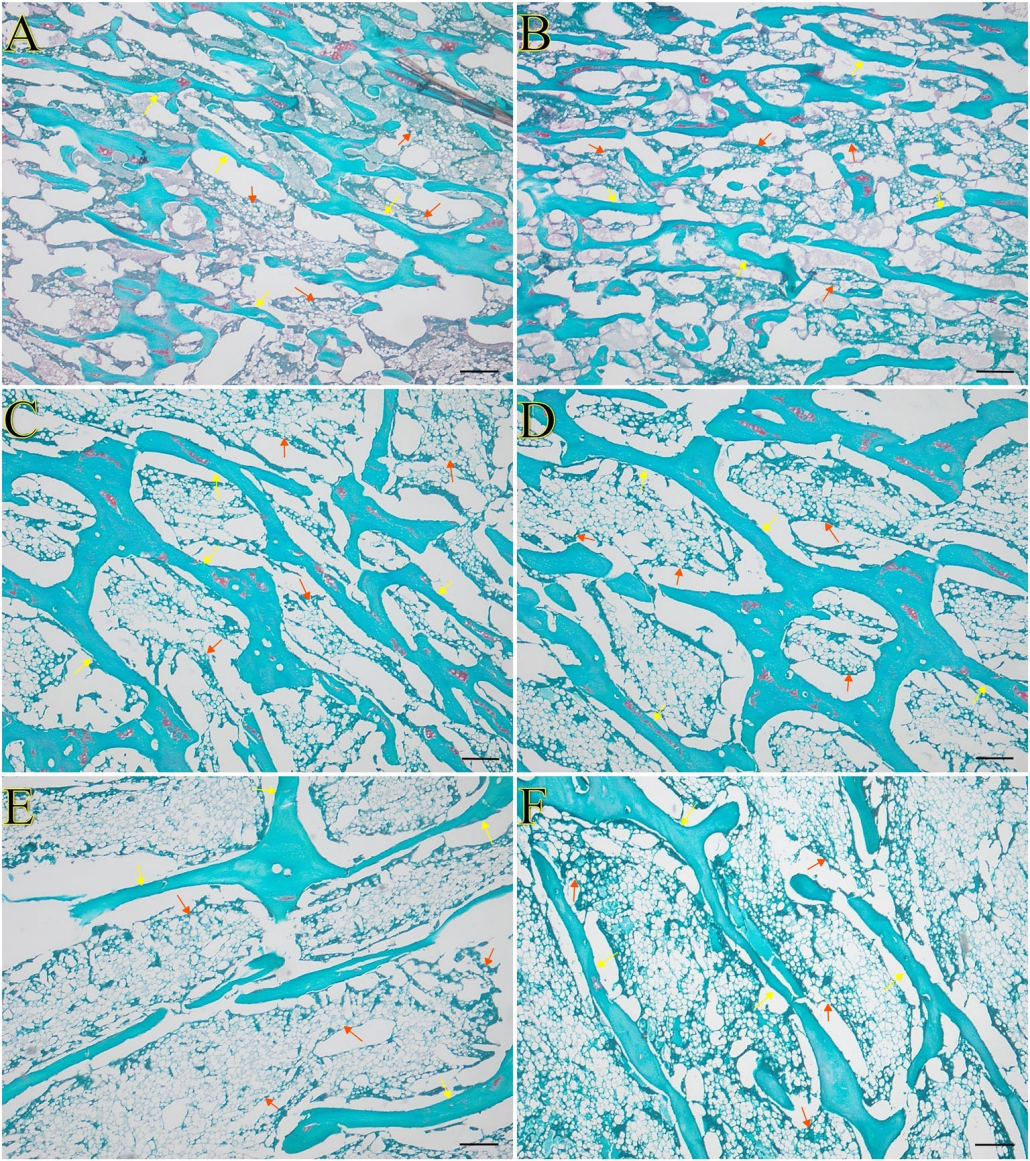
Representative images of safranin O staining of the trabecular bone of tibia bone of 16-week-old, 20-week-old and 24-week-old roosters and capons. (**A**) Roosters at 16 weeks of age, (**B**) capons at 16 weeks of age, (**C**) roosters at 20 weeks of age, (**D**) capons at 20 weeks of age, (**E**) roosters at 24 weeks of age, (**F**) capons at 24 weeks of age. The description of the groups as in the Fig. 1. Yellow arrows indicate trabeculae, red arrow indicate bone marrow. All scale bars represent 200 µm.

Histomorphometric parameters of trabecular bone in tibia are presented in Fig. 1A–D. The caponization has a significant effect on the relative bone volume BV/TV which were higher values of this parameter among the 20-week-old capons compared to roosters at the same age (Figs. 1A, 2C,D). However, in the case of 16-week-old and 24-week-old individuals the castration did not affect the BV/TV (Figs. 1A, 2A,B,E,F). Also the trabecular thickness Tb.Th was significantly higher among 20-week-old capons compared to roosters (Figs. 1B, 2C,D), while among 16-week-old capons significantly lower values were noted (Figs. 1B, 2A,B). In the group of 24-week-old animals, caponization has not caused differences in the Tb.Th (Figs. 1B, 2E,F). Analysis of other microstructural parameters of the trabecular bone showed that castration has a significant effect on the values of these parameters, but only in the oldest 24-week-old individuals. Capons were characterized with higher trabecular separation Tb.Sp (Figs. 1C, 2E,F) with a significant decrease in trabecular number Tb.N compared to roosters (Figs. 1D, 2E,F).

### Compact bone histomorphometric analysis

**Figure 3:**
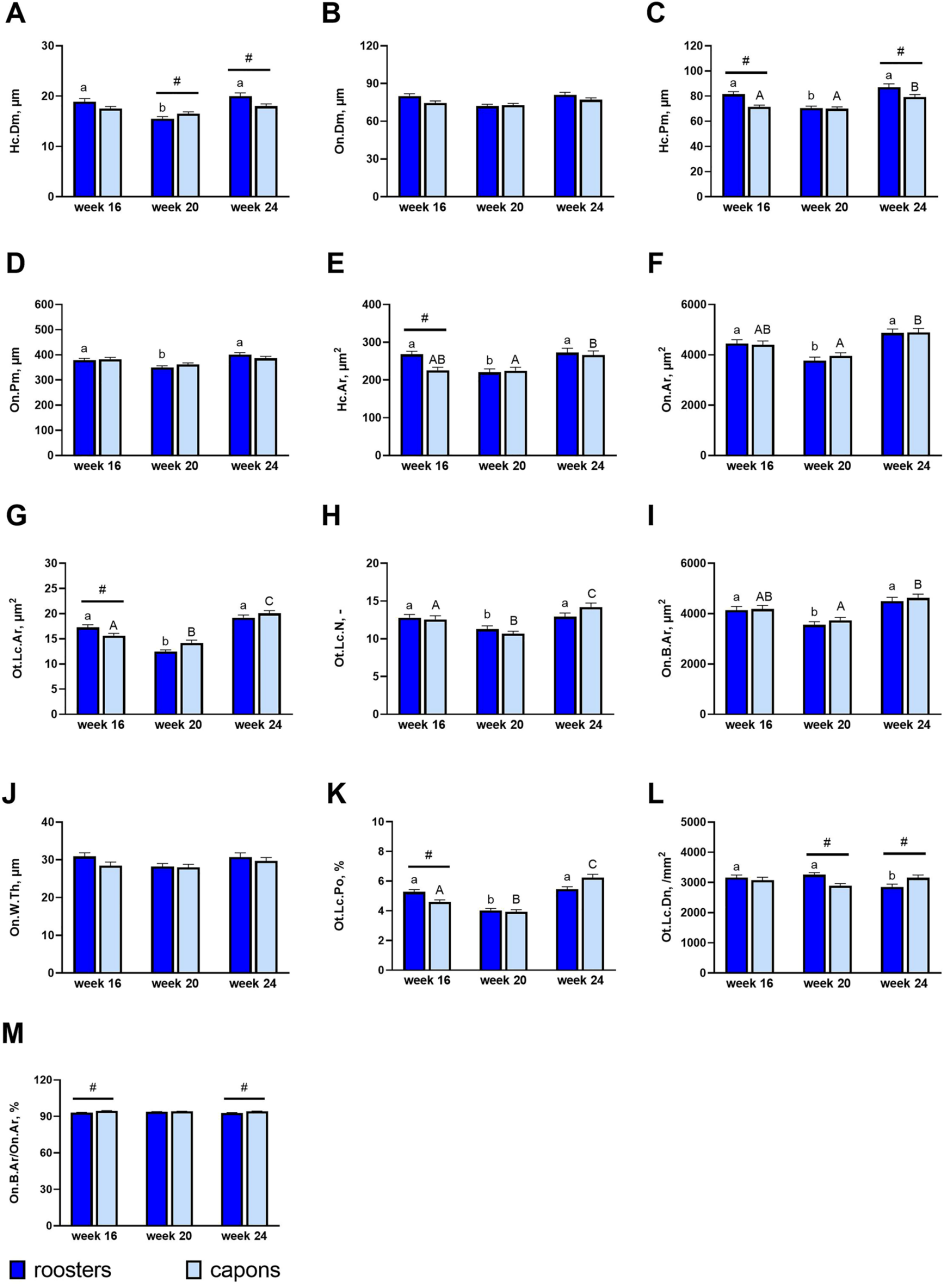
Histomorphometric parameters: Haversian canal diameter (**A**), osteon diameter (**B**), Haversian canal perimeter (**C**), osteon perimeter (**D**), Haversian canal area (**E**), osteon area (**F**), osteocyte lacunar area (**G**), osteocyte lacunar number (**H**), osteon bone area (**I**), osteon wall thickness (**J**), osteocyte lacunar porosity (**K**), osteocyte lacunar density (**L**), fraction of bone area (**M**) of the compact bone in the midshaft of tibia bone of 16-week-old, 20-week-old and 24-week-old roosters and capons. Data are presented as least squares means (LSM) and standard error of mean (SE), a, b, c—mean values between age groups within roosters with different letters differ significantly *p* < 0.05; (**A,B,C**) mean values between age groups within capons with different letters differ significantly *p* < 0.05; ^#^significant difference between capons and roosters within age (*p* < 0.05).

**Figure 4:**
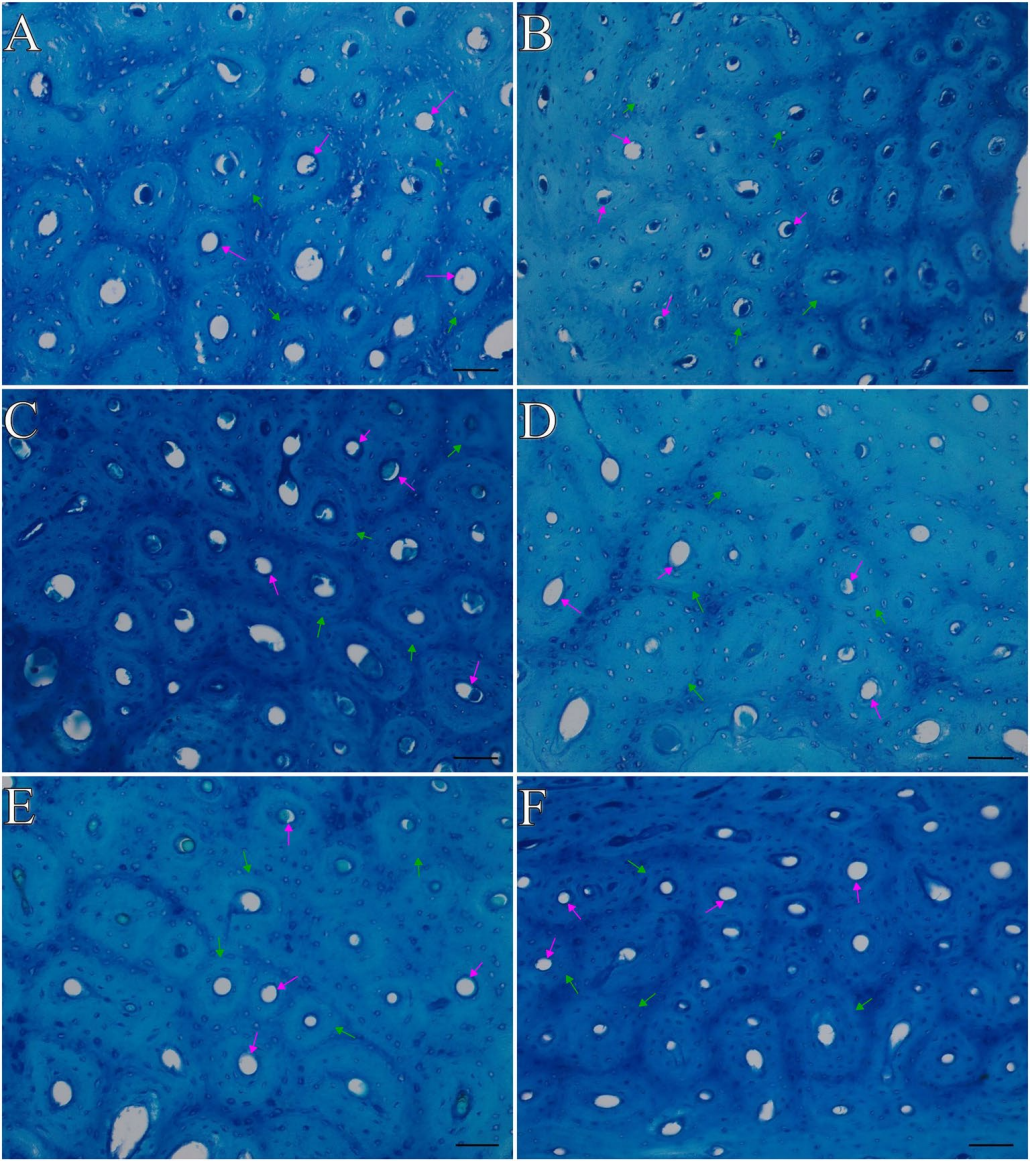
Representative images of toluidine blue staining of the compact bone of tibia bone of 16-week-old, 20-week-old and 24-week-old roosters and capons. (**A**) Roosters at 16 weeks of age, (**B**) capons at 16 weeks of age, (**C**) roosters at 20 weeks of age, (**D**) capons at 20 weeks of age, (**E**) roosters at 24 weeks of age, (**F**) capons at 24 weeks of age. The description of the groups as in the Fig. 3. Green arrows indicate osteon, violet arrow indicate Haversian canal. All scale bars represent 50 µm.

Representative microscopic images of compact bone in tibia and results of histomorphometric analysis are presented in Figs. 3A–M and 4A–F. Haversian canal diameter Hc.Dm was significantly higher in 20-week-old capons compared to roosters at the same age (Figs. 3A, 4C,D). However, among capons at 24th week of age, Hc.Dm was significantly reduced compared to roosters (Figs. 3A, 4E,F). The value of this parameter in the group of 16-week-old birds did not differ between capons and roosters (Figs. 3A, 4A,B). Caponization also caused a decrease in Haversian canal perimeter Hc.Pm in the 16- and 24-week-old individuals (Figs. 3C, 4A,B,E,F) and in Haversian canal area Hc.Ar among the 16-week-old animals (Figs. 3E, 4A,B), as well as a significantly increased fraction of bone area On.B.Ar/On.Ar in the group of 16-week-old and 24-week-old birds (Figs. 3M, 4A,B,E,F). Also the osteocyte lacunar area Ot.Lc.Ar (Figs. 3G, 4A,B) and ostecyte lacunar porosity Ot.Lc.Po (Figs. 3K, 4A,B) were lower in group of capons at 16 weeks of age compared to roosters. In the case of 20-week-old individuals, the osteocyte lacunar density Ot.Lc.Dn was significantly lower in the experimental group compared to the control group (Figs. 3L, 4C,D). However, the opposite results were observed in the group of 24-week-old birds (Figs. 3L, 4E,F). No effects of caponization on the osteon diameter On.Dm (Figs. 3B, 4A–F), osteon perimeter On.Pm (Figs. 3D, 4A–F), osteon area On.Ar (Figs. 3F, 4A–F), osteocyte lacunar number Ot.Lc.N (Figs. 3H, 4A–F), osteon bone area On.B.Ar (Figs. 3I, 4A–F) and osteon wall thickness On.W.Th (Figs. 3J, 4A–F) were noted.

### Distribution of thick and thin collagen fibers in trabecular bone

**Figure 5:**
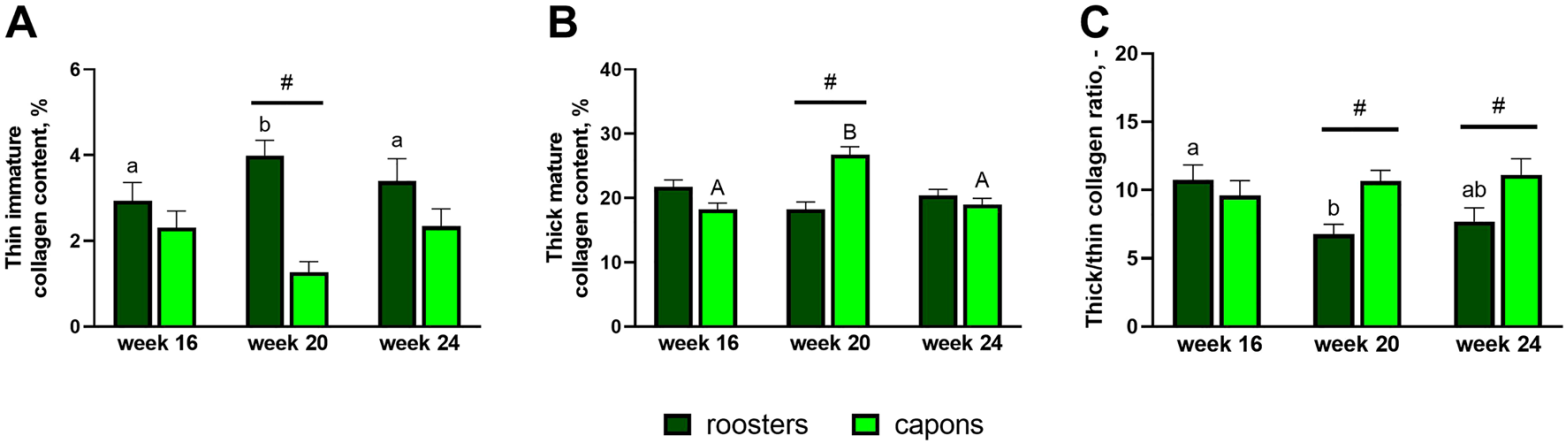
Thin immature collagen content (**A**), thick mature collagen content (**B**), thick/thin collagen ratio (**C**) in trabecular bone in the epiphyses of tibia bone of 16-week-old, 20-week-old and 24-week-old roosters and capons. Data are presented as least squares means (LSM) and standard error of mean (SE), a, b, c—mean values between age groups within roosters with different letters differ significantly *p* < 0.05; (**A,B,C**)—mean values between age groups within capons with different letters differ significantly *p* < 0.05; ^#^significant difference between capons and roosters within age (*p* < 0.05).

**Figure 6:**
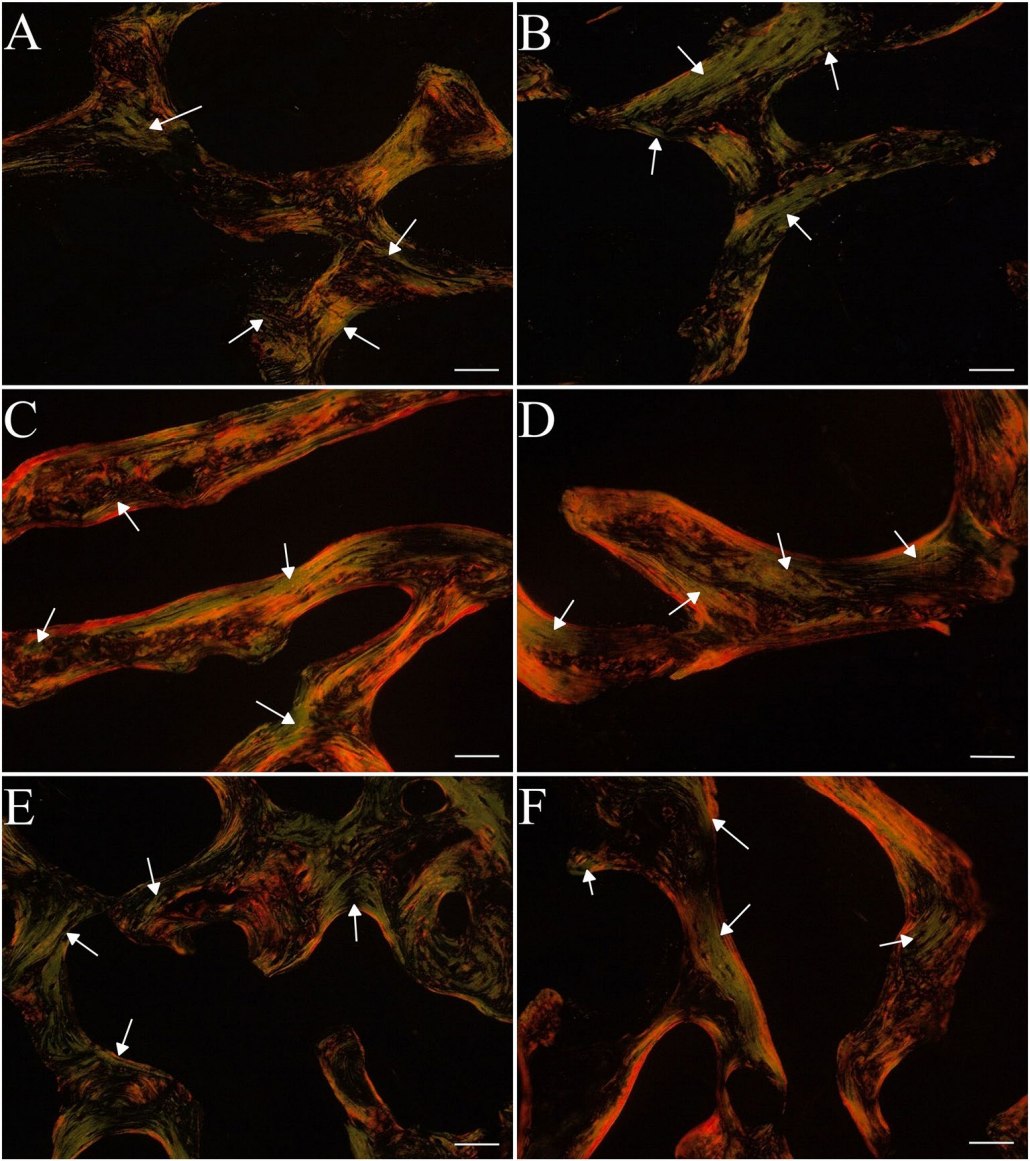
Representative images of PSR staining of the trabecular bone of tibia bone of 16-week-old, 20-week-old and 24-week-old roosters and capons. (**A**) Roosters at 16 weeks of age, (**B**) capons at 16 weeks of age, (**C**) roosters at 20 weeks of age, (**D**) capons at 20 weeks of age, (**E**) roosters at 24 weeks of age, (**F**) capons at 24 weeks of age. The description of the groups as in the Fig. 5. White arrows indicate thin immature collagen. All scale bars represent 50 µm.

In the trabecular bone the caponization increased thick-mature (red) collage content and decreased thin-immature (green) collagen content but only in the group of 20-week-old individuals (Figs. 5A,B, 6C,D). No differences in the values of these parameters were observed between capons and roosters at 16 and 24 weeks of age (Figs. 5A,B, 6A,B,E,F). Also, a significant increase in the ratio of mature to immature (thick/thin) collagen fibers was noted in 20-week-old and 24-week-old capons compared to roosters (Figs. 5C, 6C–F). In the case of 16-week-old animals, the ratio of mature to immature collagen fibers remained at the same level in the control and experimental group (Figs. 5C, 6A,B).

### Distribution of thick and thin collagen fibers in compact bone

**Figure 7:**
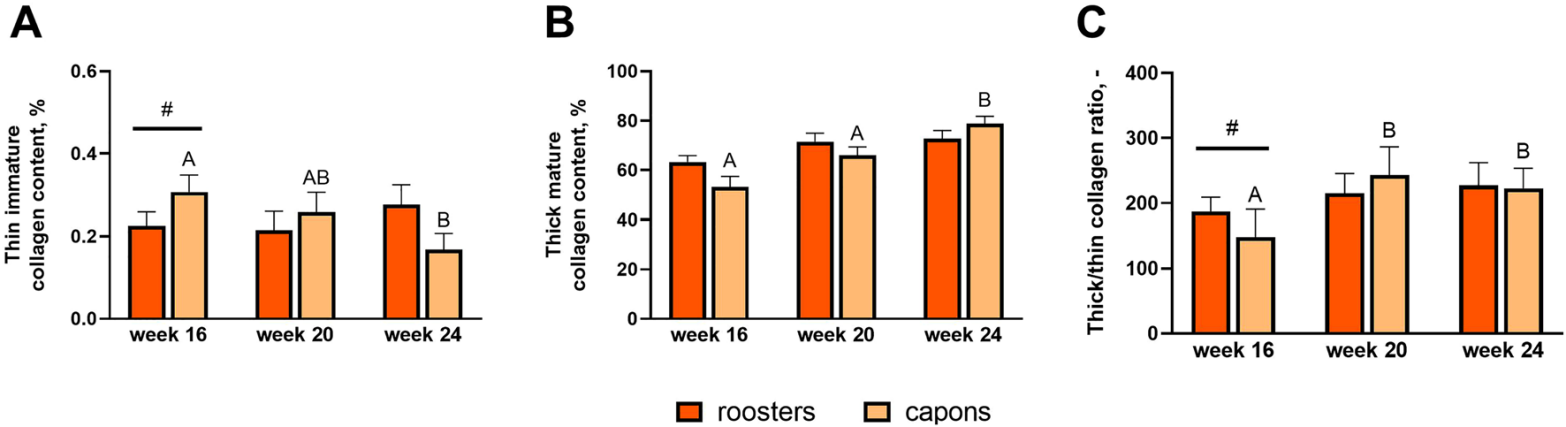
Thin immature collagen content (**A**), thick mature collagen content (**B**), thick/thin collagen ratio (**C**) in compact bone in the midshaft of tibia bone of 16-week-old, 20-week-old and 24-week-old roosters and capons. Data are presented as least squares means (LSM) and standard error of mean (SE), a, b, c—mean values between age groups within roosters with different letters differ significantly *p* < 0.05; (**A,B,C**) mean values between age groups within capons with different letters differ significantly *p* < 0.05; ^#^significant difference between capons and roosters within age (*p* < 0.05).

**Figure 8:**
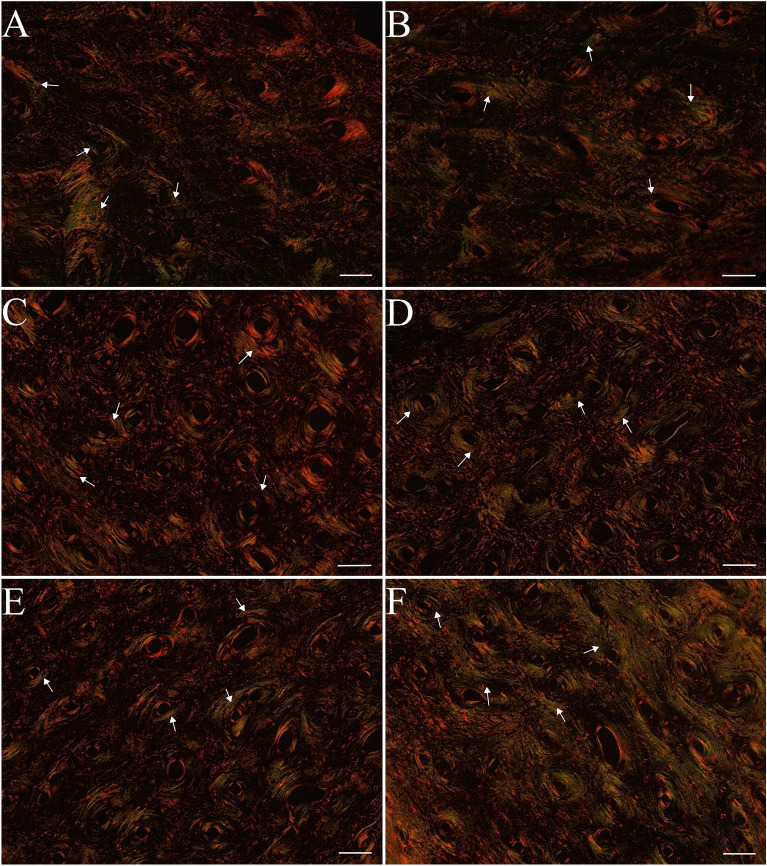
Representative images of PSR staining of the compact bone of tibia bone of 16-week-old, 20-week-old and 24-week-old roosters and capons. (**A**) Roosters at 16 weeks of age, (**B**) capons at 16 weeks of age, (**C**) roosters at 20 weeks of age, (**D**) capons at 20 weeks of age, (**E**) roosters at 24 weeks of age, (**F**) capons at 24 weeks of age. The description of the groups as in the Fig. 5. White arrows indicate thin immature collagen All scale bars represent 50 µm.

In the compact bone, the effect of caponization on collagen distribution was observed only in the group of the youngest individuals at 16 weeks of age (Figs. 7A–C, 8A–C). Among capons, a significant increase in thin-immature collagen fibers was observed with a simultaneous decrease in the ratio of mature to immature (thick/thin) collagen fibers, compared to roosters (Figs. 7A, 8A).

## Discussion

Assessment of the dynamics of metabolic processes within bone tissue are achieved by histomorphometric measurements. Due to the fact that in trabecular bone the changes occur much more intensively than in the compact bone, therefore the majority of studies are concerned with assessing the microarchitecture with the bone epiphyses. Despite the widespread use of this research method, in the available literature there is a lack of studies on the effect of caponization on histomorphometric parameters of trabecular bone. Nevertheless, numerous experiments of this type have been carried out on male rats with testosterone deficiency induced by orchidectomy. The effect of this surgery on decrease in trabecular bone mass (BV/TV) of femur and tibia was observed by many authors^25–28,30–34^. However, Libouban et al.^30^ in the case of rats, noted no changes between control and experimental groups within the 2 weeks post-orchidectomy group. The same result was obtained in a presented study in the group of 16-week-old and 24-week-old animals. On the other hand, in the case of other group, at the 20th week of age, higher BV/TV values were observed among the capons compared to roosters. In the available literature, much more varied results concern trabecular thickness. Some studies show characteristics of osteoporotic changes (trabecular thickness reduction) in the femur and tibia of castrated rats^26,28,33,34^. Additionally, some authors noted that Tb.Th was not affected by castration^30,32,35^. The same result was obtained in the presented study but only in the group of 24-week-old animals, as no effect of caponization on the value of this parameter was shown. Even more contrarily, in the group of 16-week-old individuals the decrease of Tb.Th among capons compared to roosters was observed, while an opposite effect was noted in the group of 20-week-old birds (increase Tb.Th). The weakened microarchitecture of the trabecular bone in the epiphyses is also evidenced by an increase of the trabecular separation Tb.Sp. Recent studies indicate that castration of males leads to an increase of the Tb.Sp in the epiphyses of long bones^26–28,31–34^. Similar effects were observed in the present study, but only in the group of 24-week-old individuals. However in the group of 16-week-old and 20-week-old animals, Tb.Sp did not differ between capons and roosters. In addition, the assessment of the trabecular bone microstructure is possible by measuring the trabecular number Tb.N. Many authors noted a reduction in the Tb.N in the long bone epiphyses of orchidectomized rats, which directly leads to reduction in bone quality^26–28,31–34^. The same result was obtained in the present study, as the decrease Tb.N was observed in the capons tibiae in the 24th week of their lives compared to roosters of the same age. This may indicate that castration contributes to the deterioration of the structural properties of the bones, especially in older individuals. On the other hand, Gunness and Orwoll^35^, in the case of rats, found no negative effect of castration on the Tb.N. Similarly, in our experiment in the group of younger 16- and 20-week-old individuals, caponization did not influence the value of this parameter.

As it is well known, remodelling in compact bone occurs less dynamically than in trabecular bone^36^. The histomorphometric studies of the midshaft part of long bones are rarely the subject of the research. Therefore, information on the effect of castration on histomorphometric parameters of the compact bone is limited and concerns only mammals^34,35,37^. Noteworthy, that our study is the first to analyze the effect of caponization on the midshaft tibia bone microstructure. Studies from the 1960s suggested that the size of osteons may be related to the amount of resorber bone tissue^38,39^. Some reports indicate that bone tissue remodelling is to some extent aimed at repairing microdamage to the bone^40–42^. Thus, it is suggested that different sized areas of microdamage result in different sized osteons. Therefore, the components of the osteon may also change. In the presented study, caponization has no significant effect on osteon area On.Ar, osteon perimeter On.Pm as well as osteon diameter On.Dm. However, the impact of this surgery was found within the Haversian canal. Thus, the larger Haversian canal diameter Hc.Dm was characterized by osteons of the midshaft among 20-week-old capons compared to cockerels. The opposite observations, which apply to individuals slaughtered at the 24 week of age, noted a reduction in Hc.Dm among capons compared to cockerels. On the other hand, among animals in the 16th week of life, there was no effect of castration surgery on the value of this parameter. Caponization also contributed to Haversian canal perimeter Hc.Pm decrease in individuals of 16- and 24-week-old birds and Haversian canal area Hc.Ar decrease among the youngest 16-week-old animals. It should be remembered that between lamellae there are osteocytes in a lacuna. According to Qiu et al.^43^ a larger On.Ar is associated with an increased number of osteocytes. These metabolically active cells have the ability to control the mineralization of bone tissue, bone tissue resorption and osteoid synthesis. Research conducted by Mullender et. al.^44^ show that patients with osteoporotic changes characterized increased osteocyte lacunar density Ot.Lc.Dn and number of osteocytes per unit area compared to age-matched patients from the control group. Therefore, the higher Ot.Lc.Dn within the osteon among the oldest 24-week-old capons compared to roosters in our work, may indicate an increased compact bone porosity, which could be causing microfractures within it. Thus, it can be assumed that among 24-week-old animals, caponization contributes to the weakening of the tibiotarsal midshaft, as well as it results bone loss, which is typical for osteoporosis. Calcium-deficient rats can be characterized with higher osteocyte lacunar area Ot.Lc.Ar compared to individuals from the control group^45,46^. In the presented study we noted a reduction of Ot.Lc.Ar and osteocyte lacunar porosity Ot.Lc.Po among 16-week-old capons compared to roosters. However, among 20- and 24-week-old birds no changes were observed. In addition, castration resulted in the increase of the fraction of bone area On.B.Ar/On.Ar in the case of birds in the 16th and 24th week of their rearing period. In contrast, caponization had no significant effect on osteocyte lacunar number Ot.Lc.N, osteon bone area On.B.Ar and osteon wall thickness On.W.Th.

Collagen is an important structural component of bone, formed in the regions of the bone formation. Collagen fibers are made up of type I collagen and form the main part of the bone matrix. The structure of the bone collagen does not differ from that which occurs in other connective tissues, but differs in the profile of the collagen cross-links^47^. The Picrosirus red (PSR) staining used in our work allows the visualization of thin-immature (green) type III collagen and thick-mature (red) type I collagen under polarized light^48,49^. In the available literature, there is a lack of information about the effect of caponization on the distribution of collagen fibers of the tibiotarsal bone. However, such analyses were carried out in studies assessing the influence of dietary factors on bone metabolism in poultry^50–52^. In the presented experiment the percentage of thin-immature collagen fibers, percentage of thick-mature collagen fibers and ratio of mature to immature collagen fibers were determined. Caponization, thus, reduced the level of thin-immature collagen fibers and increased the level of thick-mature collagen fibers in the tibiotarsal epiphysis of 20-week-old individuals. In addition, this surgery in the group of 20- and 24-week-old birds also increased the ratio of mature to immature collagen fibers, which may indicate a dysfunction of the collagen cross-linking in the tibiotarsal epiphysis of capons. In contrast, within the compact bone, castration contributed to the increased level of thin-immature collagen fibers and decreased the ratio of mature to immature collagen fibers but only among 16-week-old birds. According to Sparke et al.^53^ and Muszyński et al.^51^ the reduction of thin-immature collagen fibers may be related to newly synthesized collagen and thus increased turnover of bone. In contrast, Tomaszewska et al.^54^ suggests that an increase of the level of thin-immature collagen fibers may be indicative of much more intensive bone metabolism. Observations in humans indicate a higher ratio of mature to immature collagen fibers in patients with documented osteoporotic changes^55^. On this basis, it can be concluded that caponization lead to changes in collagen content particularly in the tibiotarsal epiphysis can lead to weakening of its structure and strength as the development of metabolic bone diseases.

To summarize, this is the first experiment analyzing the effect of caponization on bone microstructure of tibia bone. Our study has shown that this surgery contributes to trabeculae thinning in the tibiotarsal epiphysis of 16-week-old animals and an increase trabecular thickness among 20-week-old birds, as well as an increase trabecular separation with simultaneous decrease in the trabecular number among 24-week-old capons compared to roosters, which may indicate an intensification of resorption processes in this area in the oldest roosters. Moreover, increased bone turnover within the tibia epiphyses is also evidenced by changes in collagen fiber structure, where 20-week-old capons were characterized with lower level of thin-immature collagen fibers compared to roosters. Among 24-week-old capons there was an increase in the ratio of mature to immature collagen fibers compared to roosters, which may be a manifestation of a decrease in the rate of osteogenic processes. Additionally, this surgery contributes to changes in the compact bone microarchitecture, which is evidenced by decrease in Haversian canal perimeter and increase fraction of bone area among 16- and 24-week-old individuals, as well as increase osteocyte lacunar density of the oldest birds and changes in collagen distribution in the youngest roosters. The knowledge gained from the above study can also become a contribution to further research, primarily nutritional, aimed at improving the microstructure of capon bones.

## Methods

### Ethics approval

All procedures used in our study were approved by the 2nd Local Institutional Animal Care and Use Committee, Institute of Pharmacology, Polish Academy of Sciences in Krakow, Poland (No. 1121 of 27 November 2014). The experiment was carried out in compliance with the European Union directive no. 2010/63/EU and with the appropriate ARRIVE guidelines for reporting on experiments involving animals.

### Animals and experimental groups

The experiment was conducted on 96 hybrids obtained by crossing Rhode Island Red (R-11) cockerels and Yellowleg Partridge (${\dot{\text{Z}}}$-33) hens from conservation flock of the National Research Institute of Animal Production of Balice n. Krakow. One-day-old chicks were weighed, marked individually, and randomly assigned to two groups of 48 each. Group I (control group) consisted of intact roosters and group II (experimental group) consisted of caponised birds. The castration procedure was carried out by a veterinarian under local anesthesia (Polocaine 2%—administered subcutaneously at 1.5 cm^3^) at 8 weeks of birds’ age. Both of these groups were slaughtered at three different periods of their life (in the 16th, 20th and 24th week of their rearing period). The birds were fed ad libitum with a three-phase feeding system: phase I (1–7 weeks), phase II (8–16 weeks), phase III (17–24 weeks). Table 1 presents the results of the chemical analysis of the feed mixes according to AOAC procedures. All animals were kept in the same environmental conditions (temperature 16–18 °C, relative humidity 60–75%) in the barn system with a stocking density of 7 birds/m^2^. At the end of fattening (at 16, 20 and 24 weeks of age), 8 birds from each group with near to average body weights for each to those groups were selected for slaughter. Animals were slaughtered by decapitation after prior electrical stunning using a KOMA STZ 6 stunning apparatus (Koma, Świdnica, Poland) adapted to the species of bird and body weight gained. Before decapitation, the animals received no feed for around 12 h but had permanent access to water. Immediately post-slaughter, the castrated birds were checked for caponization success (removal of the testes) and their left tibia bones were taken. All procedures were performed in accordance with the relevant regulations and guidelines (EU Regulation No. 543/2008 of 16 June 2008 laying down detailed rules for the application of Council Regulation (EC) No. 1234/2007 as regards the marketing standards for poultry meat and EU Regulation No. 1099/2009 of 24 September on the protection of animals at the time of slaughtering).

**Table 1:** Composition and nutrient content of the diets used in the trial (kg/100 kg).

	Mixture I: 1–7 weeks	Mixture II: 8–16 weeks	Mixture III: 17–24 weeks
Ground maize	41.35	40.45	35.70
Ground wheat	25.00	22.00	29.00
Ground triticale	–	5.00	7.00
Ground barley	–	5.00	7.00
Soybean meal	30.00	24.00	18.00
Ground limestone	1.25	1.30	1.20
Dicalcium phosphate	1.60	1.45	1.30
NaCl	0.30	0.30	0.30
Vitamin-mineral premix DKA-F (finisher) (0.5%) [kg]	0.50	0.50	0.50
Crude protein [g]	193	186	163
Metabolizable energy [MJ]	11.92	12.05	12.08
[kcal]	2850	2880	2910
Lys [g]	10.3	8.90	7.50
Met [g]	3.10	2.85	2.60
Ca [g]	8.95	8.60	7.90
P available [g]	4.10	3.80	3.50

### Bone collection

After slaughter, left tibiae from individual birds were isolated and scraped away from any soft tissues. Bone samples were taken using a diamond bandsaw from the middle of the lateral tibial condyle and the middle part of tibial shaft. From the midshaft part of bone, a transverse 5 mm thick was taken and sagittal 1 mm thick fragments of proximal epiphysis were taken perpendicularly to the articular surface. All samples were fixed in 4% buffered formaldehyde for 24 h, decalcified in a commercial decalcifier (Decalcifier I, LeicaBiosystems, Nussloch, Germany), dehydrated in graded ethanol solutions (70%, 80%, 96% 100%), and embedded in paraffin. Standard histological procedures were carried out as previously reported by Dobrowolski et al.^56^ and Blicharski et al.^57^. Paraffin-fixed bone samples were cut with an RM-2145 microtome (LeicaMicrosystems, Nussloch, Germany) at a 5 µm thickness and placed on one microscopic slide. Safranin O staining was used to assess the basic histomorphometry of the trabecular bone in the proximal epiphysis of the tibia bone: relative bone volume (BV/TV), trabecular thickness (Tb.Th), trabecular separation (Tb.Sp) and trabecular number (Tb.N)^54,58^. Toluidine blue staining was used for analysis of basal histomorphometry of the cortical bone in the midshaft of the tibia bone: Haversian canal diameter (Hc.Dm), osteon diameter (On.Dm), Haversian canal perimeter (Hc.Pm), osteon perimeter (On.Pm), Haversian canal area (Hc.Ar), osteon area (On.Ar), osteocyte lacunar area (Ot.Lc.Ar), osteocyte lacunar number (Ot.Lc.N), osteon bone area (On.B.Ar), osteon wall thickness (On.W.Th), osteocyte lacunar porosity (Ot.Lc.Po), osteocyte lacunar density (Ot.Lc.Dn), fraction of bone area (On.B.Ar/On.Ar)^43,59–63^. The sections of trabecular bone and compact bone were stained with the Picrosirius red (PSR) staining giving a colour contrast of thick-mature (red) collagen fibers and thin-immature (green) collagen fibres under polarized light^64,65^. This staining was used to assess the distribution of thin collagen fibers, thick collagen fibers and ratio of mature to immature collagen fibers in trabecular and compact bone.

### Histomorphometric analysis

**Figure 9:**
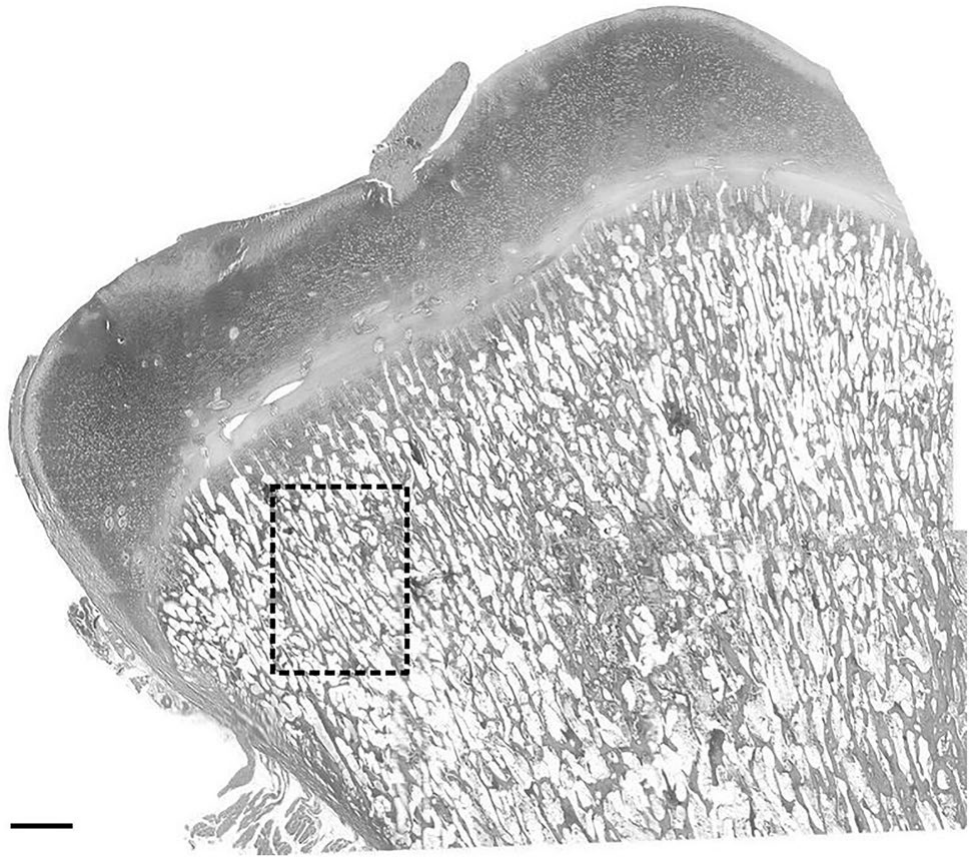
Sample schematic of the localization of histomorphometric measurements within epiphyses of tibia bone. Scale bar = 1 mm.

**Figure 10:**
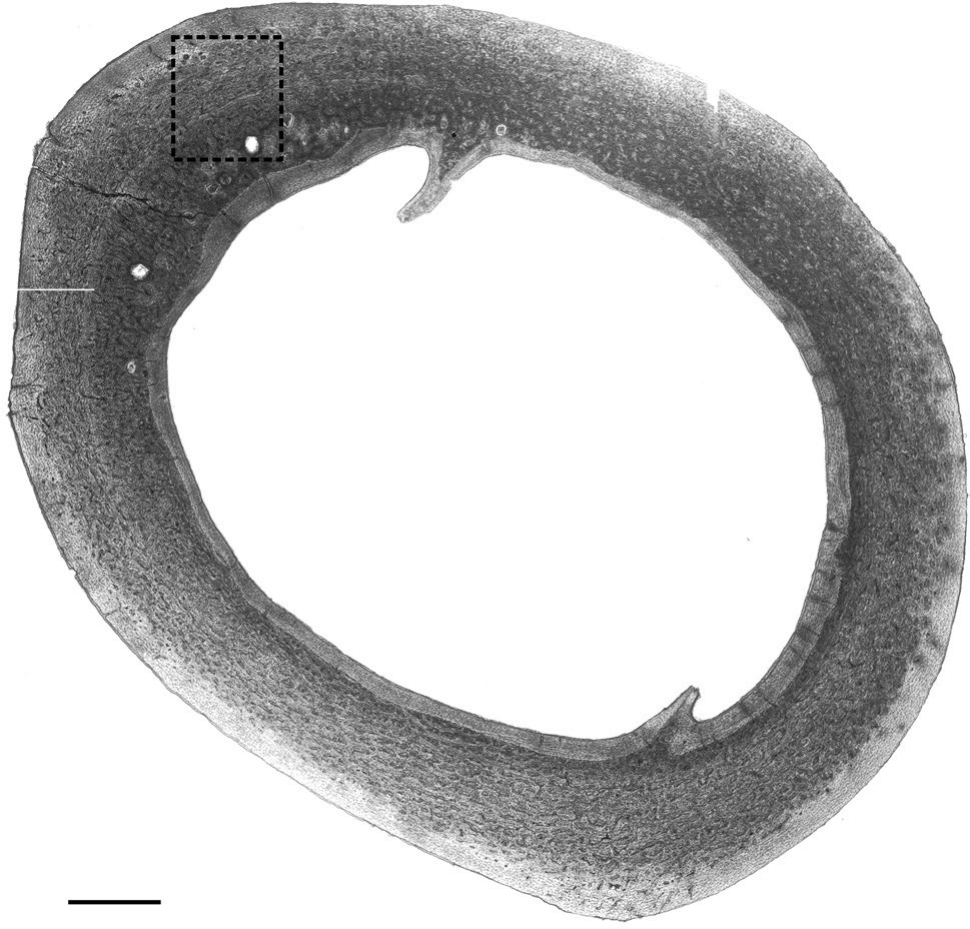
Sample schematic of the localization of histomorphometric measurements within midshaft of tibia bone. Scale bar = 0.5 mm.

Histological analysis of the prepared stained sections from the epiphysis (Fig. 9) and midshaft (Fig. 10) of tibia bone were performed using Olympus CX43 microscope (Olympus, Tokyo, Japan) and ImageJ software (NIH, Bethesda, USA).

### Reagents

All reagents were purchased from Sigma-Aldrich, St. Louis, MO, USA, unless stated otherwise.

### Statistical analysis

Calculations were performed using Statistica 13.0 (TIBCO Software Inc., Palo Alto, USA) software package. All data are presented as least-square mean (LSM) with standard error (SE). The groups (I: capons and cockerels, II: age) were compared using two-way ANOVA, applying post hoc Tukey’s test as the correction for multiple comparisons. Before testing for group differences, data were tested for normality by Shapiro–Wilk test and homogeneity of variance were tested by Brown–Forsythe test. If there was a lack of normal distribution and/or unequal variance of data, the log transformation was applied. When data still were not normally distributed the non-parametric test (Kruskal–Wallis) was used. A probability of p < 0.05 was considered to be statistically significant.

## Data Availability

The datasets presented in this study are available on reasonable request from the corresponding author.

## Abbreviations

BV/TV: Bone volume/tissue volume
Hc.Ar: Haversian canal area
Hc.Dm: Haversian canal diameter
Hc.Pm: Haversian canal perimeter
LSM: Least-square mean
On.Ar: Osteon area
On.B.Ar: Osteon bone area
On.Dm: Osteon diameter
On.Pm: Osteon perimeter
On.W.Th: Osteon wall thickness
On.B.Ar/On.Ar: Fraction of bone area
Ot.Lc.Ar: Osteocyte lacunar area
Ot.Lc.Dn: Osteocyte lacunar density
Ot.Lc.N: Osteocyte lacunar number
Ot.Lc.Po: Osteocyte lacunar porosity
SE: Standard error
Tb.N: Trabecular number
Tb.Sp: Trabecular separation
Tb.Th: Trabecular thickness

## References

[1] Sirri F, Bianchi M, Petracci M, Meluzzi A (2009). Influence of partial and complete caponization on chicken meat quality. Poult. Sci..

[2] Symeon GK, Mantis F, Bizelis I, Kominakis A, Rogdakis E (2010). Effects of caponization on growth performance, carcass composition, and meat quality of medium growth broilers. Poult. Sci..

[3] Amorim A, Rodrigues S, Pereira E, Teixeira A (2016). Physicochemical composition and sensory quality evaluation of capon and rooster meat. Poult. Sci..

[4] Lin CY, Hsu JC, Wan TC (2012). Effect of age and caponization on blood parameters and bone development of male native chickens in Taiwan. Asian Australas J. Anim. Sci..

[5] Guo X (2015). Effects of caponization on growth, carcass, and meat characteristics and the mRNA expression of genes related to lipid metabolism in roosters of a Chinese indigenous breed. Czech J. Anim. Sci..

[6] Tor M, Estany J, Villalba D, Molina E, Cubilo D (2002). Comparison of carcass composition by parts and tissues between cocks and capons. Anim. Res..

[7] Franco D, Pateiro M, Rois D, Vazquez JA, Lorenco JM (2016). Effects of caponization on growth performance, carcass and meat quality of mos breed capons reared in free-range production system. Ann. Anim. Sci..

[8] Calik J, Obrzut J (2023). Physicochemical characteristics of meat from capons derived from crossing of conserved breed hens and meat roosters. Poult. Sci..

[9] Sinanoglou VJ, Mantis F, Miniadis-Meimaroglou S, Symeon GK, Bizelis IA (2011). Effects of caponization on lipid and fatty acid composition of intramuscular and abdominal fat of medium-growth broilers. Br. Poult. Sci..

[10] Calik J, Połtowicz K, Świątkiewicz S, Krawczyk J, Nowak J (2015). Effect of caponization on meat quality of Greenleg partridge cockerels. Ann. Anim. Sci..

[11] Calik J (2014). Capon production—Breeding stock, rooster castration and rearing methods, and meat quality—A review. Ann. Anim. Sci..

[12] Kwiecień M, Kasperek K, Grela E, Jeżewska-Witkowska G (2015). Effect of caponization on the production performance, slaughter yield and fatty acid profile of muscles of Greenleg Partridge cocks. J. Food Sci. Technol..

[13] Calik J, Świątkiewicz S, Obrzut J, Połtowicz K, Krawczyk J (2020). Effect of caponization on growth performance and meat physicochemical properties of crossbred chickens. Ann. Anim. Sci..

[14] Diaz O, Rodriguez L, Torres A, Cobos A (2010). Chemical composition and physico-chemical properties of meat from capons as affected by breed and age. SJAR.

[15] Rikimaru K, Takahashi H, Nichols MA (2011). An efficient method of early caponization in slow-growing meat-type chickens. Poult. Sci..

[16] Muszyński S (2017). Effect of caponization on performance and quality characteristics of long bones in Polbar chickens. Poult. Sci..

[17] Symeon GK (2013). Effects of caponization on fat metabolism-related biochemical characteristics of broilers. J. Anim. Physiol. Anim. Nutr..

[18] Chen SY, Li TY, Tsai CH, Lo DY, Chen KL (2014). Gender, caponization and exogenous estrogen effects on lipids, bone and blood characteristics in Taiwan country chickens. Anim. Sci. J..

[19] Chen KL, Chi WT, Chiou PWS (2005). Caponization and testosterone implantation effects on blood lipid and lipoprotein profile in male chickens. Poult. Sci..

[20] Quaresma MAG (2017). Immunocastration as an alternative to caponization: Evaluation of its effect on body and bone development and on meat color and composition. Poult. Sci..

[21] Tomaszewska E (2017). Long-bone properties and development are affected by caponisation and breed in Polish fowls. Br. Poult. Sci..

[22] Kwiecień M (2019). Effect of caponisation on bone development in native male chickens. Ann. Anim. Sci..

[23] Aguado E, Pascaretti-Grizon F, Goyenvalle E, Audran M, Chappard D (2015). Bone mass and bone quality are altered by hypoactivity in the chicken. PLoS ONE.

[24] Dalle Carbonare L, Giannini S (2004). Bone microarchitecture as an important determinant of bone strength. J. Endocrinol. Investig..

[25] Erben RG, Eberle J, Stahr K, Goldberg M (2000). Androgen deficiency induces high turnover osteopenia in aged male rats: A sequential histomorphometric study. J. Bone Miner. Res..

[26] Chin K-Y, Ima-Nirwana S (2014). Effects of annatto-derived tocotrienol supplementation on osteoporosis induced by testosterone deficiency in rats. Clin. Interv. Aging.

[27] Iwamoto J, Seki A (2015). Effects combined teriparatide and monthly risedronate therapy on cancellous bone mass in orchidectomized rats: A bone histomorphometric study. Calcif. Tissue Int..

[28] Mohamad N-V, Ima-Nirwana S, Chin K-Y (2020). The effects of gonadotropin-releasing hormone agonist (buserelin) and orchidectomy on bone turnover markers and histomporphometry in rats. Aging Male.

[29] Wojciechowska-Puchałka J (2023). The effect of caponization on bone homeostasis of crossbred roosters. I. Analysis of tibia bone mineralization, densitometric, osteometric, geometric and biomechanical properties. Sci. Rep..

[30] Libouban H (2002). Comparison of histomorphometric descriptors of bone architecture with dual-energy X-ray absorptiometry for assessing bone loss in the orchidectomized rat. Osteoporos. Int..

[31] Khalil DA (2005). Soy isoflavones may protect against orchidectomy-induced bone loss in aged male rats. Calcif. Tissue Int..

[32] Soung DY (2006). Soy affects trabecular microarchitecture and favorably alters select bone-specific gene expressions in a male rat model of osteoporosis. Calcif. Tissue Int..

[33] Filipović B (2007). The effect of orchidectomy on thyroid C cells and bone histomorphometry in middle-aged rats. Histochem. Cell Biol..

[34] Iwamoto J, Yeh JK, Takeda T (2003). Effect of vitamin K2 on cortical and cancellous bones in orchidectomized and/or sciatic neurectomized rats. J. Bone Miner. Res..

[35] Gunness M, Orwoll E (1995). Early induction of alterations in cancellous and cortical bone histology after orchiectomy in mature rats. J. Bone Miner. Res..

[36] Li J (2017). Different bone remodeling levels of trabecular and cortical bone in response to changes in Wnt/β-catenin signaling in mice. J. Orthop. Res..

[37] Iwamoto J, Takeda T, Matsumoto H, Sato Y, Yeh JK (2008). Beneficial effects of combined administration of alendronate and alfacalcidol on cancellous bone mass of the tibia in orchidectomized rats: A bone histomorphometry study. J Nutr. Sci. Vitaminol. (Tokyo).

[38] Landeros O, Frost HM (1964). The cross section size of the osteon. Henry Ford Hosp. Med. Bull..

[39] Takahashi H, Frost HM (1965). The presence of frequency maxima in histograms od resorption space sizes in human rib cortex. Henry Ford Hosp. Med. Bull..

[40] Burr DB (1993). Remodeling and the repair of fatigue damage. Calcif. Tissue Int..

[41] Parfitt AM (1993). Bone age, mineral density, and fatigue damage. Calcif. Tissue Int..

[42] Bentolila V (1998). Intracortical remodeling in adult rat long bones after fatigue loading. Bone.

[43] Qiu S, Fyhrie DP, Palnitkar S, Rao SD (2003). Histomorphometric assessment of Haversian canal and osteocyte lacunae in different sized osteons in human rib. Anat. Rec. A Discov. Mol. Cell Evol. Biol..

[44] Mullender MG, van der Meer DD, Huiskes R, Lips P (1996). Osteocyte density changes in aging and osteoporosis. Bone.

[45] Sissons HA, Kelman GJ, Marotti G (1984). Mechanisms of bone resorption in calcium-deficient rats. Calcif. Tissue Int..

[46] Sissons HA (1990). A light and scanning electron miscroscopic study of osteocyte activity in calcium-deficient rats. Calcif. Tissue Int..

[47] Knott L, Bailey AJ (1998). Collagen cross-links in mineralizing tissues: A review of their chemistry, function, and clinical relevance. Bone.

[48] Lattouf R (2014). Picrosirius red staining: A useful tool to appraise collagen networks in normal and pathological tissues. J. Histochem. Cytochem..

[49] Kanai R (2020). Effects of surface sub-micrometer topography following okalic acid treatment on bone quantity and quality around dental implants in rabbit tibiae. Int. J. Implant. Dent..

[50] Muszyński S (2018). Analysis of bone osteometry, mineralization, mechanical and histomorphometrical properties of tibiotarsus in broiler chickens demonstrates a influence of dietary chickpea seeds (*Cicer arietinum* L.) inclusion as a primary protein source. PLoS ONE.

[51] Muszyński S (2020). The effect of dietary rye inclusion and xylanase supplementation on structural organization of bone consitutive phases in laying hens fed a wheat-corn diet. Animals.

[52] Tomaszewska E (2020). The effect of bee pollen on bone biomechanical strength and trabecular bone histomorphometry in tibia of young Japanese quail (*Coturnix japonica*). PLoS ONE.

[53] Sparke AJ (2002). Differences in composition of avian bone collagen following genetic selection for resistance to osteoporosis. Br. Poult. Sci..

[54] Tomaszewska E, Dobrowolski P, Puzio I, Donaldson J, Muszyński S (2020). Acrylamide-induced prenatal programming of bone structure in mammal model. Ann. Anim. Sci..

[55] Bailey AJ, Wotton SF, Sims TJ, Thompson PW (1992). Post-translational modifications in the collage of human osteoporotic femoral head. Biochem. Biophys. Res. Commun..

[56] Dobrowolski P, Tomaszewska E, Muszyński S, Blicharski T, Pierzynowski SG (2017). Dietary 2-oxoglutarate prevents bone loss caused by neonatal treatment with maximal dexamethasone dose. Exp. Biol. Med..

[57] Blicharski T, Tomaszewska E, Dobrowolski P, Hałus-Stasiak M, Muszyński S (2017). A metabolite of leucine (β-hydroxy-β-methylbutyrate) given to sows during pregnancy alters bone development of their newborn offspring by hormonal modulation. PLoS ONE.

[58] Flis M (2019). The influence of the partial replacing of inorganic salts of calcium, zinc, iron, and copper with amino acid complexes on bone development in male pheasants from aviary breeding. Animals.

[59] Skedros JG, Clark GC, Sorenson SM, Taylor KW, Qiu S (2011). Analysis of the effect of osteon diameter on the potential relationship of osteocyte lacuna density and osteon wall thickness. Anat. Rec..

[60] McGee-Lawrence ME (2011). Thirteen-lined ground squirrels (*Ictidomys tridecemlineatus*) show microstructural bone loss during hibernation but preserve bone macrostructural geometry and strength. J. Exp. Biol..

[61] Pazzaglia UE (2012). A model of osteoblast-osteocyte kinetics in the development of secondary osteons in rabbits. J. Anat..

[62] Bernhard A (2013). Micro-morphological properties of osteons reveal changes in cortical bone stability during aging, osteoporosis, and bisphosphonate treatment in women. Osteoporos. Int..

[63] Hunter RL, Agnew AM (2016). Intraskeletal variation in human cortical osteocyte lacunar density: Implications for bone quality assessment. Bone Rep..

[64] Tomaszewska E (2017). The effect of tannic acid on bone mechanical and geometric properties, bone density, and trabecular histomorphometry as well as the morphology of articular and growth cartilages in rats co-exposed to cadmium and lead is dose dependent. Toxicol. Ind. Health.

[65] Ida T (2018). Extracellular matrix with defectiva collage cross-linking affects the differentiation of bone cells. PLoS ONE.

